# Recent advances in understanding and managing pediatric inflammatory bowel disease

**DOI:** 10.12688/f1000research.19609.1

**Published:** 2019-12-13

**Authors:** Bhaskar Gurram, Ashish S. Patel

**Affiliations:** 1 University of Texas Southwestern Medical Center, Dallas, TX, 75390, USA

**Keywords:** pediatric inflammatory bowel disease, Crohn’s disease, ulcerative colitis

## Abstract

The landscape of pediatric inflammatory bowel disease is rapidly evolving. The therapeutic advances seen in the adult arena are rapidly being adopted by pediatric gastroenterologists and evaluated in both controlled trials and real-world experience. Though anti-tumor necrosis factor agents have been the primary therapy over the last decade, recently there has been an expansion of therapeutic targets and alternative mechanism of action drugs with a focus on individualized and personalized therapy. By reviewing epidemiology, pathophysiology, and goals of treatment, we hope to frame the discussion of current and novel therapeutics for the pediatric gastroenterologist. As scientific discovery continues to push the envelope in defining our understanding of pediatric inflammatory bowel disease, the current era of therapeutics gives us hope that a cure may be realized soon.

## Introduction

The landscape of pediatric inflammatory bowel disease (IBD) is rapidly evolving. The therapeutic advances seen in the adult arena are rapidly being adopted by pediatric gastroenterologists and evaluated in both controlled trials and real-world experience. Additionally, pediatrics is leading the way in several key areas including the understanding of IBD as a monogenic disease in early onset (less than 10) and very early onset (less than 6) cases and around diet as primary therapy utilizing regimens including the specific carbohydrate diet (SCD) and exclusive enteral nutrition (EEN). Just a short 10 years ago, primary therapy in pediatric IBD centered around steroids and oral immunomodulators. With the advent of the first anti-tumor necrosis factor alpha (TNF-α) inhibitor, we were ushered into the biologic era. Anti-TNF agents were more efficacious drugs with the potential for sustained remission. Pediatric gastroenterologists began discussing the potential for an effective therapy like an anti-TNF to change the natural history of this previously aggressive and progressive disease. Though anti-TNFs were the main drug to occupy this class for almost a decade, recently there has been an expansion of therapeutic targets and alternative mechanism of action drugs with a focus on individualized and personalized therapy.

As we continue to increase our understanding of therapeutics, the natural evolution of thought shifts from merely modulating the body’s inflammatory response to actually curing the disease. Most current Venn diagrams of pediatric IBD pathophysiology intersect genetic susceptibility, environmental triggers, host immune response, and the gut microbiome resulting in IBD. A key driver, particularly in pediatrics, is the concept that in young children this may be more of a monogenic than a multifactorial disease, as described above. Work by investigators like Dr. Scott Snapper and consortiums like the InterNational Early Onset Paediatric IBD Cohort Study (NEOPICS) has identified unique monogenic immune defects previously classified under the IBD nomenclature that are actually unique defects that have an IBD-like presentation and represent monogenic defects that mimic IBD. These diseases respond and can be cured by treatments like bone marrow transplantation rather than the traditional immunosuppression used in IBD.

The understanding of pediatric IBD has also been greatly influenced by the development of the Improve Care Now (ICN) network, led by Dr. Richard Colletti. The network began with 12 centers in 2007, named the Trailblazer Consortium, and has evolved to 107 centers worldwide all pushing the envelope of quality care, resulting in standardized evaluation, consistent therapeutic dosing, focus on nutrition and growth, and, in the end, sustained steroid-free remission. The ICN network has developed clinical pathways, which we will highlight in the appropriate areas, in addition to consistently contributing to the pediatric literature on optimal IBD care.

## Epidemiology

The incidence and prevalence of Crohn’s disease (CD) and ulcerative colitis (UC) varies widely across the globe, and there is a paucity of data from many developing countries. Overall, it appears that the rates of pediatric IBD are increasing globally in both developed and developing countries; however, accurate estimates are lacking ^1^. Based on the reports, there appear to be variations within a country based on geographic location. For example, in the USA, the incidence in Texas was lower compared to the incidence in Wisconsin (2.4 versus 9.5 per 100,000 population, respectively) ^2, 3^. Population-based health administrative data from Canada showed an incidence of pediatric IBD to be 9.68 per 100,000 population, which means Canada has one of the highest incidences of pediatric IBD in the world ^4^. In addition, this study did not find a change in the overall incidence for the duration of study between 1999 and 2010. However, there was an increase in incidence in children between 0 and 5 years of age.

Racial and geographic differences in the incidence have been well described. In British Columbia, South Asians had a threefold higher incidence of pediatric IBD than non-South Asians, and they had a distinct phenotype with more extensive colonic disease and severe disease compared to the non-South Asian population ^5, 6^. Asian and Hispanic children develop UC more often than CD ^7^. A higher prevalence with increasing latitudes has been noted for pediatric-onset CD in Scotland ^8^ and for UC in Finland ^9^. In a population-based study in Europe, the combined incidence rates for CD and UC in all western European centers were twice as high as the rates in the Eastern European centers, implicating a geographic variation in IBD incidence ^10^.

## Advances in the understanding of pediatric IBD pathophysiology

IBD is thought to be of multifactorial origin with complex interaction between genes and environment. Twin studies and family studies have suggested a strong genetic component to IBD pathogenesis and incidence ^11^. Although having a first-degree relative with IBD confers a greater risk (risk of CD to first-degree relatives of CD patients was 10-fold greater and the risk of UC to relatives of UC patients was eightfold greater than the risk of CD or UC to first-degree relatives of healthy controls) than any known environmental factor, the rate at which the incidence has increased worldwide over the past century ^12^ and more recently in Asia ^13^ significantly exceeds that which can be explained by a genetic drift alone. Genome-wide association studies have identified over 200 genetic loci associated with IBD ^14^. These genes are implicated in immune homeostasis and the regulation of innate functions like response to microbiota, autophagy, endoplasmic reticulum stress response, and mucosal barrier integrity. These loci are also implicated in adaptive immune function ^15^, yet combined these explain only 15 to 26% of heritability for IBD. Moreover, these genetic variations are population specific, and more than 70% of these genetic loci are shared with other autoimmune diseases like type 2 diabetes and rheumatoid arthritis.

The gut microbiome is thought to play a significant role in the pathogenesis of IBD. Studies have shown alterations in the microbiome in IBD patients compared to healthy controls with decreases in diversity and abundance. However, there are significant inconsistencies regarding the results, potentially from how the microbiome studies were conducted and interpreted. It remains unclear whether microbiome changes lead to intestinal inflammation or change as a result of it.

The true interaction between genes and the environment is very difficult to discern and likely involves a complex multidirectional interplay mediated by the epigenome, microbiota, and the innate and acquired immune system.

## Goals of treatment

IBD, especially CD, is an aggressive and progressive illness that can lead to irreversible bowel damage. With the introduction of biologics, the approach to the management of IBD has changed from controlling symptoms which do not necessarily correlate with inflammation to achieving mucosal healing a more objective measure. Thus, over the past two decades, there has been a shift in the treatment goal from relief of symptoms to inducing mucosal healing while continuing to maintain growth, support nutrition, improve quality of life, and minimize side effects. Preliminary support for this paradigm shift came from the first large trial of infliximab (IFX), ACCENT-I. Patients who achieved mucosal healing were less likely to have CD-related hospitalizations and surgery ^16^. Furthermore, using mucosal healing as an end point for decision making was found to be more cost-effective than a strategy based on clinical symptoms by decreasing disease-related complications ^17^.

There are several other markers in the management of IBD. These include CRP, albumin, fecal calprotectin, and antibodies against various antimicrobial antigens ^18^. Although elevated CRP correlates well with both endoscopic and histologic evidence of inflammation, in patients with isolated small bowel CD and those with UC the correlation with disease activity is poor ^19^. Similarly, although calprotectin is considered a good marker of intestinal inflammation, especially in UC, it does not correlate well with CD activity, especially small bowel CD ^18^.

There are several reports discussing the role of serological markers in IBD management. One study involving a large pediatric multicenter cohort demonstrated that disease progression from uncomplicated to internal penetrating or stricturing disease phenotypes and CD-related surgery is accelerated in the presence of antimicrobial immune reactivity that included anti-Cbir1 (flagellin), anti-outer membrane protein C antibody (anti-ompC), anti- *Saccharomyces cerevisiae* antibody (ASCA), and perinuclear anti-neutrophil cytoplasmic antibody (pANCA). Both the number of immune responses and the magnitude of immune response to various microbial antigens were predictive of aggressive disease phenotypes. The group positive for all three antibodies and those patients with the highest magnitude of response (the highest quartile sum group 4) exhibited the most rapid disease progression ^20^. The odds ratio (OR) for the development of internal penetrating disease was 5.0 and 9.5 for children with reactivity to two and three antigens, respectively. Another cross-sectional study involving an adult population demonstrated that patients who were positive for ASCA IgA and IgG were 8.5 times and 5.5 times more likely to undergo early (<3 years) surgery than patients negative for ASCA IgA and IgG ^21^.

Several studies have suggested that nucleotide-binding oligomerization domain 2 ( *NOD2*) mutations are associated with an increased risk of complicated CD. The *NOD2* gene codes for NOD 2 protein, which is an intracellular pattern recognition receptor primarily involved in recognizing muramyl dipeptide, a molecule present on certain bacteria, and helps in modulating the immune system. In a meta-analysis, Adler *et al.* reported that the presence of a single *NOD2* mutation predicted an 8% increase in the risk for complicated disease (B2 or B3) and a 41% increase with two mutations ^22^. Although the predictive power associated with a single *NOD2* mutation for complicated disease was weak (relative risk: 17%), the presence of two *NOD2* mutations had 98% specificity for predicting complicated disease ^22^. However, *NOD2* mutations were poor predictors for postoperative recurrence of CD ^23^.

Kugathasan *et al.* derived a risk stratification model for complicated disease behavior based on clinical, serological, gene expression pattern, and microbiota data on 913 treatment-naïve pediatric CD patients ^24^. These patients were prospectively followed for about 36 months and it was found that about 9% (78) of patients had complicated disease course. This risk stratification model had an area under the receiver operator characteristic curve of 0.72, sensitivity of 69%, specificity of 71%, positive predictive value of 24%, and negative predictive value of 94%. Older age at diagnosis, African American race, and ASCA and CBir1 sero-positivity were associated with disease complications; early anti-TNF-α therapy was associated with a reduction in penetrating disease, and an ileal extracellular matrix gene signature at diagnosis was associated with stricturing disease.

Serological markers (ASCA, pANCA, anti-cbir1, anti-flagellin, etc.) and genetic markers are rarely used in clinical practice, as their applicability is limited by their limited sensitivity and the added cost.

Seigel *et al.* developed and validated a tool (Personalized Risk and Outcome Prediction Tool [PROSPECT]) to predict an individual patient’s risk of developing a CD complication based on clinical, serologic, and genetic variables ^25^. The PROSPECT tool generates an individualized risk based on the information provided and groups the patients into low, medium, and high risk for surgery in 3 years and could help physicians and patients on personalized treatment options.

## Exclusive enteral nutrition

EEN has been used for several decades in the induction of remission in patients with CD. It is more widely prescribed in Canada and the European nations than in the US. EEN involves supplying 100% of one’s caloric needs as a formula, polymeric or hydrolyzed, enterally for 8–12 weeks. The remission rates in most cohort studies range from 60 to 80% ^26– 28^, which is equivalent to corticosteroid remission rates. When compared to corticosteroids, which are also used for the induction of remission, EEN is as effective at decreasing symptoms and biochemical markers of inflammation (ESR and CRP) but, unlike steroids, it also leads to decreased endoscopic severity grossly and histologically ^28, 29^. When compared to corticosteroids, EEN is also associated with improved linear growth, bone mineralization, and lean body mass acquisition as opposed to fat mass acquisition with corticosteroids; additionally, it is not immunosuppressive ^30, 31^. Therefore, EEN can be particularly helpful in patients with significant growth failure or malnutrition.

European and North American guidelines recommend EEN as the first-line agent in active luminal CD diagnosed under 17 years of age ^32, 33^. Although early data indicated that EEN was more likely effective in patients with small bowel involvement ^34^, subsequent data have shown effectiveness regardless of the site of involvement ^27^. However, efficacy has not been demonstrated in perianal, fistulizing, or stricturing CD.

In a recent meta-analysis, Swaminathan *et al.* demonstrated that EEN is equally efficacious in inducing remission in newly diagnosed (OR = 1.61 [95% CI 0.87, 2.98]) and relapsed (OR = 0.76 [95% CI 0.29–1.98]) patients ^35^. In this study, the likelihood of achieving mucosal healing was higher with EEN compared to steroids. Biomarker normalization did not differ.

The exact mechanism of action has remained uncertain, but various mechanisms have been proposed, including decreased antigen exposure, alteration of the resident microbiome ^36^, restoration of epithelial barrier integrity, and decreased immune system activation ^37^.

## Specific carbohydrate diet and other diet-based therapies

The SCD was first developed in the 1920’s by Dr. Sydney Haas as a therapy for celiac disease and later popularized by a mother of one of Dr. Haas’ patients, Elaine Gottschall, a biochemist who wrote the book *Breaking the Vicious Cycle: Intestinal Health Through Diet.* The diet is a nutritionally balanced one that removes grains, dairy, processed foods, and sugars, except for honey. The theory is that these items pass undigested or poorly digested into the distal intestine, allowing the overgrowth of harmful bacteria. This has been re-energized recently by the work of Dr. David Suskind at Seattle Children’s who has published several small case series showing the efficacy of the SCD in treating pediatric IBD ^38– 41^. The diet is being evaluated nationally in a trial called PRODUCE led by the ICN network centers.

The CD exclusion diet (CDED) is an exclusion diet supplemented with partial enteral nutrition. This involves eliminating certain foods that are deemed to induce dysbiosis, specifically gluten, dairy products, gluten-free baked goods and breads, animal fat, processed meats, products containing emulsifiers, canned goods, and all packaged products with an expiration date. CDED has been studied and found to be beneficial in both children and adults with IBD. In a recent randomized study that included 78 pediatric patients with mild-to-moderate CD, CDED was found to be better tolerated and had higher rates of remission compared to EEN ^42^. Other less-well-studied diets in IBD include ordinary food-based diets that replicate EEN (CD-TREAT) ^43^, the food influence on Intestinal MicrobioTa diet ^44^, the anti-inflammatory diet ^45^, and low-fermentable oligosaccharide, disaccharide, monosaccharide, and polyols (low FODMAP) diet ^46^.

## 5-aminosalicylic acid (5ASA) compounds

5-ASA compounds are one of the earliest compounds introduced for the management of IBD. Sulfasalazine was the only 5-ASA formulation shown to have modest benefit compared to placebo in mild-to-moderate CD restricted to the colon ^47^. They are not found to be of any benefit in small bowel CD or in moderate-to-severe CD and should not be used ^48– 50^ in these patients. ECCO and ESPGHAN guidelines recommend 5-ASA compounds as first-line agents for the induction and maintenance of mild-to-moderate UC ^51^. Combination of oral and rectal 5-ASA therapy is considered superior to 5-ASA monotherapy ^52^. The ICN Model IBD Care document recommends oral dosing of 60 to 100 mg/kg/day in active disease and 30 to 100 mg/kg/day in maintenance phase. In a randomized controlled study of mesalamine, there was no difference in the efficacy of once versus twice daily dosing for mild-to-moderate pediatric UC ^53^; thus, once-daily dosing is encouraged to improve patient compliance. The overall efficacy as measured by steroid-free remission at 1 year with mesalamine ranges from 32 to 45% ^51^. Sulfasalazine was found to be superior to other 5-ASA medications but also had increased side effects ^54, 55^. It is estimated that up to 20 to 25% of patients discontinue sulfasalazine because of side effects. The most common side effects encountered with sulfasalazine include nausea, headache, fever, and rash. Curcumin (an active component of turmeric) 3 g/day with mesalamine was found to be more effective than mesalamine alone in achieving clinical and endoscopic remission in patients with mild-to-moderate UC without an increase in adverse effects ^56^.

## Thiopurines

Azathioprine (AZA) and 6-mercaptopurine (6-MP) have traditionally been the first-line immunomodulators used for the maintenance of remission in CD and UC. However, data concerning long-term risks with thiopurines including skin cancer, lymphoma, and hepatosplenic T-cell lymphoma, particularly in young males receiving thiopurines alone or in combination with anti-TNF-α antibodies, have led pediatric gastroenterologists to favor methotrexate (MTX) in children with CD. Results from the multicenter pediatric IBD network (PIBDNet) showed that 69% of patients (45 of 65 patients) achieved remission within 180 days of thiopurine initiation. However, only 47% and 23% remained in sustained steroid-free remission at 6 and 12 months, respectively. There was significant variation in the dose of thiopurine used, and there was variation in serum 6-TG levels ^57^.

AZA is a prodrug and is converted non-enzymatically to 6-MP. Although AZA and 6-MP could be used interchangeably, the dosing is different. The recommended dose for AZA is 1.5 to 2.5 mg/kg per day orally (maximum dose 200 mg per day), and 6-MP is generally given at doses of between 1 and 1.5 mg/kg per day orally (maximum 150 mg per day). To determine a safe starting dose, thiopurine methyltransferase genotype (TPMT genotype) or phenotype (TPMT activity) testing is recommended prior to beginning treatment with 6-MP or AZA. TPMT is an enzyme that is essential in the metabolism of thiopurines. Approximately 90% of patients are homozygous for the allele TPMT 1 (TPMT 1/1 genotype) and have normal or rapid metabolism of thiopurines. In contrast, 10% of patients have reduced TPMT activity (TPMT 1/3a genotype) and metabolize thiopurines more slowly. These patients typically respond to much lower doses of the medication (0.5 mg/kg/day of 6-MP). Approximately 1 in 300 patients has absent TPMT activity (TPMT 3a/3a genotype), does not metabolize the medication, and can develop agranulocytosis ^58, 59^.

However, in a systematic review, thiopurine metabolite testing did not safely predict clinical outcome but may facilitate toxicity surveillance and treatment optimization in poor responders ^60^. Current evidence favors the combination of thiopurine metabolites, WBC and aminotransferase monitoring for prompt identification of hematologic and hepatic toxicity, safe dose adjustment, and treatment modification in cases of suboptimal clinical outcome or non-compliance ^60– 62^. All patients should have regular clinic follow-up with documented lymph node exam and abdominal palpation for hepatic or splenic enlargement.

Although thiopurines may be effective in prolonging response and remission ^63^, adverse side effects may reduce their utilization. In one series that looked at 95 children and adolescents with IBD who received AZA or 6-MP, 54% had no adverse effect, 28% experienced side effects necessitating dose reduction (elevated liver transaminases, leukopenia, and lymphopenia), and 18% required drug discontinuation because of pancreatitis, recurrent fever, nausea and/or vomiting, and recurrent infections such as sinusitis ^64^.

A newer formulation of 6-MP is being developed that is locally active and is a delayed-release formulation with minimal systemic absorption. In a phase II study, Israeli *et al.* reported that in CD when compared to systemically delivered 6-MP (Purinethol), delayed-release 6-MP (DR-6MP) showed similar efficacy, a greater proportion of remission with low systemic bioavailability, and a low incidence of side effects ^65^.

Owing to the published risk of hemophagocytic lymphohistiocytosis (HLH) with primary EBV while on thiopurines, there is a growing practice of screening all newly diagnosed children for EBV status and, if naïve, avoiding all thiopurine use.

## Methotrexate

MTX is an anti-folate agent that is used for induction and maintenance of remission in CD ^66, 67^. It is not useful in the management of UC ^68– 70^. Most of the evidence for efficacy in pediatric patients comes from several retrospective pediatric series, a recent systematic review ^71^, and extrapolated information from one randomized placebo-controlled trial in adults with CD ^72^. The pooled achieved clinical remission rate for pediatric CD patients on monotherapy within 3–6 months was 57.7% (95% CI 48.2–66.6%) ( *P* = 0.22; I ^2^ = 29.8%). The clinical remission was 37.1% (95% CI 29.5–45.5%) ( *P* = 0.20; I ^2^ = 37.4%) for maintenance therapy at 12 months ^71^. In general, MTX given orally has significant variability in bioavailability among individuals; subcutaneous injection is the preferred formulation ^73^. The superiority of subcutaneous MTX compared to oral MTX is well demonstrated in rheumatology literature ^73^.

MTX has dose-limiting side effects including hepatotoxicity and bone marrow suppression. In one systematic review that included 12 high-quality studies evaluating hepatotoxicity (defined as elevated liver transaminases) when used in a pediatric IBD population, Valentino *et al.* described hepatotoxicity in 1 in 10 patients, 1 in 15 patients needed dose reduction, and 1 in 22 patients needed discontinuation of MTX. Based on these findings, the authors recommended monitoring liver biochemistries at baseline, every 2 weeks for the first month, and then every 2–3 months ^74^. Although there is insufficient literature looking at the utility of folate supplementation with MTX in the IBD population, it is known to be beneficial in patients with rheumatoid arthritis. In rheumatoid arthritis, folate supplementation (1 mg/day) with MTX is known to reduce the risk of side effects including liver transaminase elevation and gastrointestinal side effects like nausea and vomiting ^75, 76^. Folate supplementation also promoted compliance and reduced patient withdrawal compared to placebo ^76^.

## Anti-TNFs

IFX, a chimeric monoclonal antibody against TNF-α, is the most commonly used biologic in the treatment of children with CD and UC. Standard IFX dosing in children is 5 mg/kg at 0, 2, and 6 weeks during induction followed by maintenance doses of 5 mg/kg every 8 weeks.

The REACH trial ^77^ is a landmark study showing the efficacy of IFX in children for induction and maintenance of remission in moderate-to-severe CD. In this study of 112 children with CD, approximately 60% of patients had clinical response or remission at 1 year. Subsequent studies have shown that up to 50% of patients would need dose modification by 54 weeks to retain or regain efficacy ^78– 80^. Following maintenance therapy initiation, the likelihood of continuing maintenance IFX at 1, 2, and 3 years was 93%, 78%, and 67%, respectively ^80^.

In moderate-to-severe active pediatric UC that failed conventional therapy (5-ASAs, corticosteroids, or immunomodulators), IFX was found to be safe and effective, inducing a response at week 8 in 73.3% ^81^. However, the overall remission rate at week 54 for all enrolled patients was 28.6% ^81^.

Current evidence indicates that the treatment failure may result in part from low IFX serum levels. IFX trough levels of <3 mg/ml are associated with worse clinical outcomes, and dose optimization based on trough levels has proven beneficial in some studies ^78, 82, 83^. Factors contributing to the observed pharmacokinetic differences include BMI and sex, inflammatory burden (extent and severity of disease), serum albumin, and presence or absence of a concomitant immunomodulator and of anti-drug antibodies (neutralizing antibodies) ^84^. Among these factors, weight and serum albumin levels have been found to have the largest influence ^84^. In one pediatric study looking at the correlation of serum IFX levels at week 14 as predictors of remission at week 54, the authors found that trough levels of at least 3 mg/ml were associated with a positive predictive value of only 76% for persistent clinical remission at week 54 ^78^. Increasing the trough threshold to at least 7 mg/ml improved the positive predictive value of persistent remission at week 54 to 100% ^78^.

Using the data from 112 patients with CD in the REACH trial, Frymoyer *et al.* constructed a Monte Carlo simulation analysis of hypothetical children with CD to address dose optimization of IFX based on weight and albumin levels. They found that standard IFX maintenance dosing of 5 mg/kg every 8 weeks is predicted to frequently result in trough concentrations of <3 mg/ml in children with CD with an albumin of 4 g/dl or less ^85^. They estimated that only 21% or 41% of children would achieve trough concentrations of >3 mg/ml if albumin levels were 3 g/dl or 4 g/dl, respectively, and thus a substantial proportion of patients would need an increase in dose and frequency to maintain adequate drug exposure at week 14 ^85^.

Thus, early IFX trough levels could help in guiding dose optimization and likely improve clinical outcomes. Although therapeutic drug monitoring (TDM), defined as the evaluation of levels of drug and anti-drug antibodies, has proven to be effective in optimizing anti-TNF therapy in maintenance phase of IBD, the studies are limited to monitoring levels in induction phase. However, logically thinking, it is likely that there is a beneficial effect of monitoring levels during induction phase and optimizing them rather than waiting until the maintenance phase. Based on preliminary data in adults with UC, week 2 and week 6 trough levels of 30 to 36 and 24 to 30 mg/ml at week 2 and 6, respectively, were associated with early mucosal healing ^86^. In children with moderate-to-severe UC, week 8 levels of serum IFX concentrations (≥41.1 μg/ml) were associated with greater proportions of patients achieving efficacy endpoints (clinical response: 92.9%; mucosal healing: 92.9%; and clinical remission: 64.3%) versus those with lower serum concentrations (<18.1 μg/ml; 53.9%, 53.9%, and 30.8%, respectively) ^87^. A retrospective study from a tertiary care center demonstrated that interval shortening rather than dose escalation results in higher IFX levels, and the authors recommended a dose of every 6 weeks for optimizing levels ^88^.

Based on the above data, we recommend checking IFX trough levels before the fourth dose (week 14 dosing) in CD to guide subsequent intervals between dosing and achieve target levels of >5 mg/ml. For IFX in severe UC, our practice has been to start with 10 mg/kg, in particular in patients with documented hypoalbuminemia. For UC, we recommend checking levels before the third (week 6 dosing) induction dose with levels of >20 mg/ml and adjusting the subsequent doses accordingly.

Adalimumab, a human IgG monoclonal antibody against TNF-α, was approved for use in children with CD in 2012. The standard recommended dosing in children weighing >40 kg is 160 mg and 80 mg for induction 2 weeks apart and 40 mg every 2 weeks for maintenance. For children weighing <40 kg, induction dosing is 80 mg and 40 mg 2 weeks apart and 20 mg every 2 weeks for maintenance. In the pivotal study IMAgINE 1, using adalimumab in moderate-to-severe CD, 33.3% of patients remained in remission at week 54. Patients who were naïve to IFX had higher remission rates (45% versus 19%) ^89^. About 50% of patients in the IMAgINE 1 trial needed dose escalation to weekly dosing from every other week dosing based on clinical symptoms; this frequent dosing was well tolerated ^90^. The IMAgINE 2 study assessed the long-term efficacy through week 240 of adalimumab ^91^. At week 240, about 41% and 48% of those enrolled achieved remission and response as assessed by PCDAI<10 and PCDAI decrease by >15 points from baseline, respectively ^91^. In a systematic review that included 14 studies (one randomized controlled trial and 13 case series) and 664 patients, the pooled remission rates were 44% (n = 169/383) at 12 months. Of the total patients, 6% (n = 13/207) were classified as primary non-responders and 12% (n = 69/599) had severe adverse events reported, including two deaths and one medulloblastoma ^92^.

Based on a network meta-analysis of five randomized controlled trials in moderate-to-severe adult UC, adalimumab was comparable in efficacy to IFX at 52 weeks of maintenance treatment ^93^. Based on the retrospective studies, adalimumab in pediatric UC showed similar results to those of adults with UC ^94^. While on adalimumab, reactive TDM is recommended in adults, with a recommended minimum drug concentration at week 4 of >7 mg/ml to achieve mucosal healing and levels of >5 mg/ml during maintenance phase ^95, 96^.

Golimumab, another human IgG monoclonal antibody against TNF-α, is approved for use in adults with UC. In an open-label pharmacokinetic study of 35 children with moderate-to-severe UC, golimumab showed a week 6 clinical response in 60%, with mucosal healing achieved in 23%, and clinical remission in 57% at week 14 ^96, 97^. The recommended dosing in children with UC for induction is 200 mg at week 0 and 100 mg at week 2 if body weight is >45 kg and 120 mg/m ^2^ at week 0 and 60 mg/m ^2^ at week 2 if body weight is <45 kg. Maintenance dosing is 100 mg every 4 weeks for >45 kg body weight and 60 mg/m ^2^ every 4 weeks if body weight is <45 kg ^96, 97^. In adults, the expected minimum concentration at week 6 for golimumab should be >2.5 mg/ml, and maintenance trough levels should be >1 mg/ml ^95^.

Safety issues of TNF-α medications include acute infusion reactions (within 4 hours of IFX infusion), delayed hypersensitivity reactions (beyond 4 hours and up to 14 days), and serious and opportunistic infections. In one retrospective study that included a total TNF-α exposure of 390.5 patient-years (PYs), the overall incidence rate of serious adverse events (SAEs) for IFX was 22.49/100 PYs ^98^. The most common SAEs were anaphylactoid reactions (n = 18) followed by infectious events (n = 9) and TNF-α antagonist-induced lupus-like syndrome (n = 3). The overall incidence rate of SAEs for adalimumab was 4.71/100 PYs (two infectious SAEs). No malignancies or deaths were observed in this cohort. When used as monotherapy TNF-α medications are not associated with increased risk of malignancy or HLH in the pediatric population ^99^. However, in the adult population, there is increased risk of malignancies even with monotherapy, with the highest risk associated with combination therapy with thiopurines ^100^. Psoriasis has been well documented as an adverse class effect of TNF-α, but it is usually mild and controllable in most patients with topical therapy ^101^.

There is no clear evidence that pre-medication with IFX prevents the development of acute infusion reaction ^102^, and we do not routinely use or recommend the use of pre-medications prior to IFX infusion. Recommended infection screening before starting TNF-α medications includes screening for tuberculosis (PPD, quantiferon TB, or chest X-ray), hepatitis B (Hep B surface antigen and antibody), and varicella immunization status (confirmed vaccination, exposure, or checking for varicella IgG) ^51^.

## Combination therapy

The use of an immunomodulator drug, either thiopurine (AZA or 6-MP) or MTX, with TNF-α antibody is termed combination therapy. Demonstrated benefits of combination therapy include a reduction in the formation of antibodies to TNF-α and increased durability of biologic response ^103^. However, the benefits on clinical remission and mucosal healing are unclear. Some of the risks to consider in using combination therapy are increased risk of infections and increased risk of malignancy.

The SONIC trial comparing the efficacy of AZA, IFX, and AZA+IFX that included 508 patients with CD naïve to AZA and IFX showed higher steroid-free remission and mucosal healing with combination of AZA and IFX ^104^. Combination therapy with AZA appears to improve efficacy by increasing the pharmacokinetic features of IFX ^105^. Based on these data, the American Gastroenterological Association (AGA) recommended using combination therapy for induction of remission in adults with CD ^106^.

Data on combination therapy in pediatric CD comes from one randomized controlled trial and several retrospective studies and the results are inconclusive, with some studies showing no benefit of combination therapy ^107– 111^ and others showing benefit with regard to remission and durability of response to combination therapy ^112– 116^. One therapeutic option is to use low-dose immunomodulators in combination with TNF-α antibodies ^113, 117^.

Two important safety concerns with the use of combination therapy are increased risk of infections and malignancy. Two of the important risk factors for increased risk of infection in IBD patients were identified to be moderate-to-severe disease and concomitant steroid use. A clinical report on combination therapy by the North American Society for Pediatric Gastroenterology, Hepatology, and Nutrition (NASPGHAN) concluded that based on the adult literature, the use of combination therapy may have a net effect of reducing infectious risk in select patients by improving disease remission rates and minimizing steroid use ^118^.

With regard to malignancy risk, especially with lymphoma, the risk is primarily attributed to thiopurines and there may be a slight increased risk when anti-TNFs are used in combination ^99, 119^. One exception is hepatosplenic T cell lymphoma (HSTCL). An increased incidence of HSTCL has been reported in young male patients treated with either thiopurines as monotherapy or in combination with a TNF-α ^120, 121^.

Taking the above benefits and risks into consideration, there certainly is a group of patients who benefit from using combination therapy to induce remission and prevent long-term risks associated with CD while at the same time minimizing the side effects of combination therapy. At our center, we typically use combination therapy in patients with moderate-to-severe CD, especially with stricturing or fistulizing disease and severe perianal disease. We prefer to use MTX over thiopurines in males and those with no evidence of EBV infection in the past. We also use lower doses of immunomodulators when used in combination with TNF-α drugs.

## Anti-adhesion agents

Anti-adhesion molecules act by blocking the trafficking of T-lymphocytes from the lymphoid organs and bloodstream to the site of gut inflammation. There are two medications in this group. Natalizumab was the first anti-adhesion agent approved for the management of moderate-to-severe CD. Natalizumab is a monoclonal antibody against the α4 integrin on lymphocytes. Specifically, it blocks the α4β7 and α4β1 integrins on the T cells from binding mucosal addressin cell adhesion molecule-1 (MADCAM-1) and vascular cell adhesion molecule-1 (VCAM-1), respectively ^122^. Natalizumab, in comparison to placebo, has been reported to be more effective in the induction and maintenance of remission in patients with CD ^123^. The evidence in pediatrics is limited to two case series ^124, 125^. Hyams *et al.* included 31 adolescents with severe CD and reported a response rate of 55% and remission of 29% at 10 weeks ^124^.

Natalizumab’s use has been drastically limited by its association with progressive multifocal leukoencephalopathy (PML), a life-threatening CNS infection caused by the reactivation of John Cunningham virus (JCV). This side effect is thought to be directly related to the blockade of lymphocyte migration to the CNS by natalizumab. The risk factors implicated in PML include the presence of positive anti-JCV antibodies, prior immunosuppressant therapy, and prolonged duration of natalizumab therapy (>2 years). Although natalizumab was withdrawn from the US market in 2005, it was reintroduced in 2008 under restricted usage in patients with none of the risk factors mentioned above ^123^.

The second anti-adhesion biologic is vedolizumab. Vedolizumab is a monoclonal antibody against α4β7 integrin on lymphocytes, blocking their binding to MADCAM-1 and thus migration of the lymphocytes into the gastrointestinal tract mucosa. As opposed to natalizumab, vedolizumab is gut specific and does not lead to systemic immunosuppression and thus the side effects were found to be very similar to those seen with placebo ^126^. Vedolizumab has been found to be superior to placebo for induction of remission at week 6 for adult-onset CD and UC subjects with moderately to severely active disease ^126– 130^. In a single-center prospective observational study, Conrad *et al.* ^131^ followed 21 pediatric patients (13 to 21 years) with severe TNF-α-refractory IBD (16 CD, three UC, and two IBD-U). They reported a response rate in 57.9% of patients at 22 weeks with steroid-free remission of 20% at 22 weeks. Hamel *et al.* ^132^, in a retrospective cohort of 12 TNF-α and corticosteroid-refractory IBD patients (10 UC and two CD), reported that when vedolizumab used in combination with tacrolimus 9 of 12 (75%) patients avoided colectomy or IBD-related surgery at 24 weeks and 8 out of 12 (68%) continued on vedolizumab maintenance with no adverse events up to 80 weeks. A retrospective multicenter study from the pediatric IBD Porto group of ESPGHAN included 64 children with IBD (41 UC/IBD-U and 23 CD) refractory to TNF-α medications and reported a week 14 corticosteroid-free remission of 37% in UC and 14% in CD ^133^. Similarly, in a retrospective multicenter study from the US involving 52 IBD patients (58% CD and 42% UC), the authors reported a 14 week remission rate of 76% in UC and 42% in CD ^134^. The dose of vedolizumab used in children is 6 mg/kg with a maximum dose of 300 mg, with induction given at 0, 2, and 6 weeks followed by maintenance every 8 weeks ^134, 135^. The recently released VARSITY study (Sands *et al.*, DDW 2019) showed vedolizumab to have superior efficacy versus adalimumab for moderate-to-severe UC. This was a phase IIIb randomized double-blind study of 769 adults with moderately to severely active UC who had failed other therapies. Clinical remission at week 52 was the primary endpoint and was 31% with vedolizumab compared to 22% with adalimumab, and patients treated with vedolizumab had higher mucosal healing rates. Adverse events were similar in the two groups. This is an important study because it is the first comparative effectiveness study in IBD comparing two biologic therapies head to head. As opposed to natalizumab, vedolizumab has an excellent safety profile. The most frequently reported adverse events are minor and include headaches, fever, arthralgias, and nausea ^126, 127, 129, 136^. Infusion reactions are low at 3.5 per 1,000 infusions ^136, 137^.

Etrolizumab is a humanized monoclonal antibody against the β7 subunit of α4β7 and αEβ7 which prevents their interaction with MADCAM-1 and E-cadherin, respectively, and thus disrupts leukocyte migration into intestinal tissue ^123^. Etrolizumab has been shown to be well tolerated and effective in a phase II clinical trial of patients with moderate-to-severe UC ^138^; phase III clinical trials are underway. There are no pediatric studies of Etrolizumab to date.

Other medications in this group include AJM300, an orally active, small molecule with a mechanism of action like natalizumab, and PF-00547659, a monoclonal antibody administered subcutaneously against intestinal epithelial adhesion molecule MADCAM-1 ^123^.

## Interleukin inhibitors

Interleukin-12 (IL-12) and interleukin-23 (IL-23) are pro-inflammatory cytokines that induce the differentiation of naïve CD4 ^+^ T cells into T-helper 1 and T-helper 17 cells, respectively, and are important in the pathogenesis of IBD. Ustekinumab is a human monoclonal antibody against the p40 subunit that is common to both IL-12 and IL-23, consequently preventing the interaction of these cytokines with their receptors on naïve T cells and preventing further downstream signaling and the pro-inflammatory cascade. It was approved for the treatment of moderate-to-severe CD in adults in 2016.

The adult UNITI-1 and UNITI-2 studies demonstrated clinical response in adults with TNF-α-naïve and TNF-α-exposed moderate-to-severe CD patients. Responders from UNITI-1 and UNITI-2 were enrolled in a subcutaneous ustekinumab maintenance trial (IM-UNITI), which showed a clinical remission at week 44 of 53.1% in the group that received 90 mg every 8 weeks compared to 48.8% in those receiving ustekinumab every 12 weeks and 35.9% in those receiving placebo ^139^. The rates of adverse events were not statistically different between the treatment and placebo arm in all of these trials. Also, ustekinumab is observed to lead to significant reductions in endoscopic disease activity at week 8 of induction therapy ^140^.

There is an evolving body of literature for the use of ustekinumab in pediatric patients. This consists of case reports, one case series, and a recently published real-world experience on ustekinumab in pediatric patients. In the case series, the suggested dosing is 6 mg/kg IV infusion for induction followed by 45 mg every 8 weeks for maintenance in children weighing <40 kg and 90 mg every 8 weeks for children weighing >40 kg. In the real-world experience, 52 patients receiving ustekinumab were evaluated and included baseline characteristics and predictors of response. The study concluded that ustekinumab monotherapy is possible and preferable in children. A caveat that we have also noted was the need for dose amplification, 57% in this real-world experience ^141– 143^. Based on our personal unpublished experience, 8 out of 10 patients with anti-TNF-refractory disease responded to ustekinumab but needed an augmented dosing schedule of every 4 to 6 weeks during maintenance to maintain a recommended trough level of >4.5 mg/ml.

The second agent in this group is risankizumab, a humanized IgG1 monoclonal antibody that selectively binds with high affinity to the IL-23 p19 subunit and is currently being studied for use in patients with IBD. By specifically targeting the IL-23-mediated inflammatory pathway and thus the T-helper 17 cells without disrupting the IL-12-dependent T-cell pathway, which is thought to be important for infection and cancer immunity, risankizumab theoretically may confer fewer side effects in comparison with ustekinumab ^123^.

## JAK/STAT inhibitors

Janus kinases (JAKs) are intracellular signaling molecules that, when activated by cytokines, lead to phosphorylation and dimerization of Signal Transducer and Activator of Transcription proteins (STATs), which migrate into the nucleus and induce further gene transcription that modulates inflammation that is characteristic of IBD ^144^. The JAK/STAT pathway regulates intracellular signaling involving common gamma chain-containing cytokine receptors for interleukins 2, 4, 7, 9, 15, and 21 ^145^. These cytokines and their downstream JAK/STAT signaling pathways are integral to lymphocyte activation, function, and proliferation. Inhibition of the JAK/STAT signaling pathway is a major area of interest in the development of novel IBD medications ^146^.

Tofacitinib is an oral, small-molecule inhibitor of JAK1, JAK3, and, to a lesser extent, JAK2. In adults with UC, the rate of remission at 8 weeks was significantly higher in the 10 mg tofacitinib group than in the placebo group in the OCTAVE Induction 1 trial (18.5% versus 8.2%) and in the OCTAVE Induction 2 trial (16.6% versus 3.6%). In the OCTAVE Sustain trial, remission at 52 weeks occurred in 34.3% of patients in the 5 mg tofacitinib group and 40.6% in the 10 mg tofacitinib group compared to 11.1% in the placebo group ( *P* <0.001 for both comparisons with placebo). Side effects, especially rates of serious infection, were higher with tofacitinib than with placebo. However, in the OCTAVE Sustain trial, the rate of serious infection was similar across the three treatment groups, but the rates of overall infection and herpes zoster infection were higher with tofacitinib than with placebo ^147^. Other side effects noticed include dyslipidemia (high HDL and LDL cholesterol) and increased risk of cardiovascular events and non-melanoma skin cancers ^145^. In a post hoc analysis of data from the OCTAVE trials, Hanauer *et al.* ^148^ reported significant improvements in stool frequency and rectal bleeding in UC patients within the first 3 days of induction therapy with tofacitinib compared with placebo. There are no data on the use of tofacitinib in pediatric patients, and it had poor efficacy in adults with CD ^123^.

Filgotinib is a second agent in this group and is a highly selective JAK-1 inhibitor. In phase II clinical trials, filgotinib has been shown to be effective in CD and may be particularly useful in those with previous anti-TNF exposure ^149^ ( Table 1).

**Table 1.** Medication, dosing, therapeutic drug monitoring, and references.

Medication	Recommended dose for induction	Recommended dose for maintenance	Recommended trough levels for biological remission (μg/ml)	Recommended trough levels for mucosal healing (μg/ml)	Reference
Infliximab	5 mg/kg on week 0, 2, and 6	5 mg/kg every 8 weeks	3–7	>5	78,95
Adalimumab	<40 kg: 80 mg and 40 mg at week 0 and 2 >40 kg: 160 mg and 80 mg at week 0 and 2	<40 kg: 20 mg every 2 weeks >40 kg: 40 mg every 2 weeks	>5.9	>7.5	95,96
Golimumab	<45 kg: 120 mg/m ^2^ on week 0 and 60 mg/m ^2^ on week 2 >45 kg: 200 mg on week 0 and 100 mg on week 2	<45 kg: 60 mg/m ^2^ every 4 weeks >45 kg: 100 mg every 4 weeks	>1.4	NA	95
Vedolizumab	6 mg/kg on week 0, 2, and 6 (max 300)	6 mg/kg every 8 weeks (max 300)	>13.6	NA	95
Ustekinumab	<40 kg: 6 mg/kg 40 to <55 kg: 260 mg 55 to <85 kg: 390 mg >85 kg: 520 mg	<40 kg: 45 mg every 8 weeks >40 kg: 90 mg every 8 weeks	>0.8	>4.5	95

## Conclusion

Therapeutic discovery continues to teach us the many intricate and complex pathways of the human inflammatory response in IBD. We must be vigilant to the short-term and long-term effects of all of these medications, even with the new generation of gut-specific therapies. The identification of lymphoma associated with the TNF-α agents was recognized in post marketing surveillance. However, we must always frame the risk of these medications to our patients in the broader context of the natural history of this aggressive and progressive disease. As scientific discovery continues to push the envelope in defining our understanding of pediatric IBD, the current era of therapeutics gives us hope that a cure may be realized in the not-so-distant future.
